# An elbow exoskeleton for haptic feedback made with a direct drive hobby motor

**DOI:** 10.1016/j.ohx.2020.e00153

**Published:** 2020-10-22

**Authors:** Hubert Kim, Alan T. Asbeck

**Affiliations:** Mechanical Engineering Department, Virginia Tech, Blacksburg, VA 24061, USA

**Keywords:** Brushless DC motor control, Direct drive actuator, Haptic feedback, Joint torque feedback, Exoskeleton

## Abstract

A direct drive motor is one of the simplest mechanisms that can be used to move a mechanical joint. In particular, a brushless direct current (BLDC) motor with no gearing produces a low parasitic torque due to its backdrivability and low inertia, which is ideal for some applications such as wearable systems. While capable of operating with a higher power density than brushed motors, BLDC motors require accurate position feedback to be controlled via vector control at slow speeds. The MotorWare™ library from Texas Instruments (TI), which is designed to run with a C2000 microcontroller, is written to run BLDCs. However, the code was written to run the motor continuously with an incremental encoder and requires further engineering to be used at low speeds such as in an exoskeleton. In this paper, we present the design of an elbow exoskeleton that can be used for haptic feedback. We provide instructions to build the exoskeleton hardware, custom code to modify software provided by TI so that a motor can provide a controlled torque at low speeds, code to enable the microcontroller to communicate with a computer for high-level commands and data storage, and also provide an overview of how alternate motors could be used with this software setup.

## Graphical abstract

**Figure:**
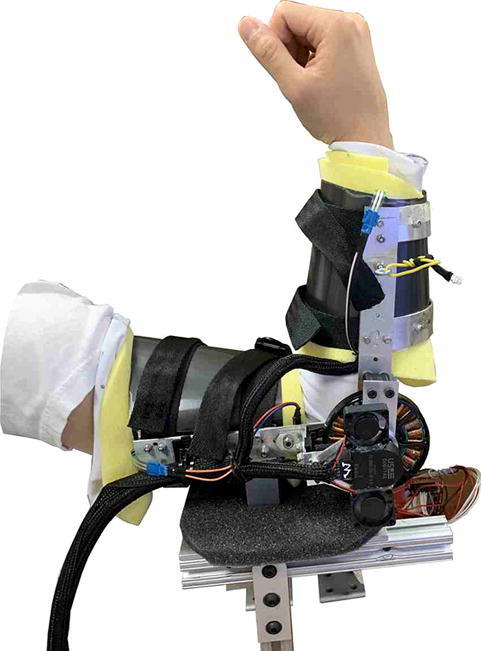

## 1 Hardware in context

There are numerous applications for an actuator that can provide controlled torques at slow speeds, including robots, test fixtures, or force feedback systems. In this paper, we focus on a wearable force feedback system (an exoskeleton) that can provide a torque to a person’s joint. Such a system can be used for motion training for sports, rehabilitation, or skills acquisition [1]. The exoskeleton presented in this paper is designed for applications where the device does not overpower the wearer but rather delivers a small torque to train a person in how to move [2].

**Table:** 

**Specifications table:**	

**Hardware name**	Elbow Haptic Exoskeleton
**Subject area**	Mechatronics
**Hardware type**	Joint actuator
**Open source license**	Berkeley Source Distribution (BSD) license
**Cost of hardware**	USD $509
**Source file repository**	https://doi.org/10.17632/skm88ynyhv.4

For rehabilitation or haptic feedback applications, the “transparency” of an exoskeleton to its wearer is an important design factor. Delays between a person’s motion and the exoskeleton’s response can cause parasitic resistances, which can hinder the wearer’s natural motion. Typically, researchers address resistance torques by using a closed-loop system where the interaction forces between the human and exoskeleton are sensed, and then the actuators are moved to reduce unwanted forces (e.g. in Harmony [3] and MGA [4]). However, even good control systems can have parasitic resistance torques of 0.3–1 Nm [3], [5], [6], which somewhat penalizes the device’s transparency, and which is much more than the just noticeable difference (JND) joint torque for haptic feedback applications [7], [8]. An alternate approach is to use a large-diameter direct drive motor (no gear train) to move a joint, which has minimal inertia and can respond to inputs quickly [1], [9], [10], [11], [12]. While the peak torque is smaller than other methods due to the lack of a gearbox, the resistance torques are close to zero. We use this approach for an elbow exoskeleton, presented in this paper, so the system can be used for haptic feedback experiments such as measuring the JND [7]. If desired, the system presented in this paper can be adapted to use a small gear ratio in order to increase the torque output, at the cost of transparency.

Kinesthetic feedback with our exoskeleton could be delivered during many possible activities, including sports, teleoperation, force feedback (e.g., in virtual reality), or motor recovery for neurologic patients, in addition to fundamental haptic feedback experiments as we have done [7]. For all of these applications, the exoskeleton’s role is to convey information through a torque or to guide the wearer to a new joint angle, similar to a trainer pushing on the arm with their hands. The paper describes the mechanical design of our exoskeleton, the microcontroller software needed to drive it, and the computer software used to provide high-level commands to the exoskeleton and log data. These modules can be easily be adapted to a wide range of other applications.

## 2 Hardware description

**Fig. 1:**
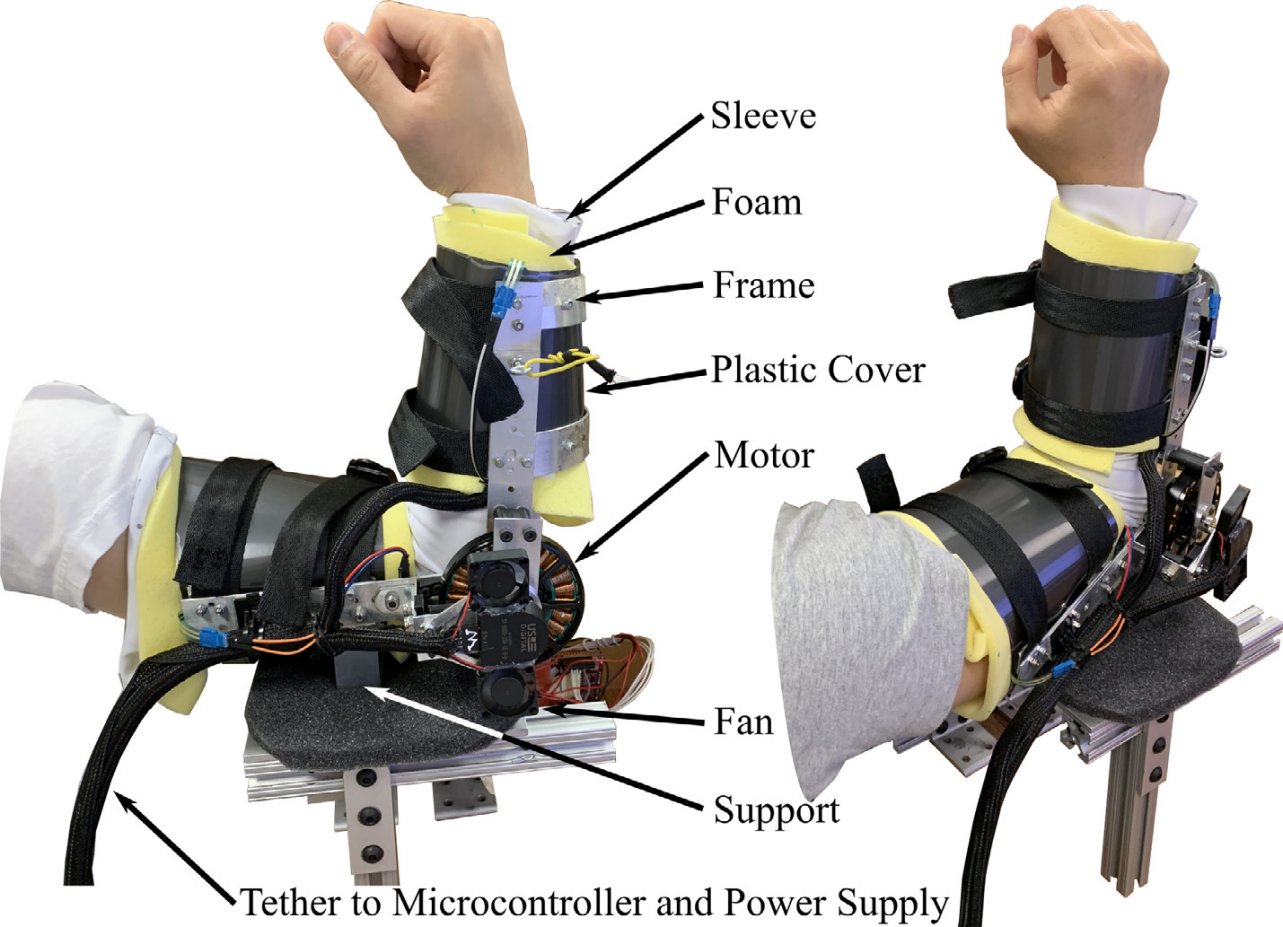
Left: side view of an arm wearing the exoskeleton. Right: Isometric view of the arm with the exoskeleton. Subjects wear the sleeve and the foam, followed by tightening the straps. Aluminum support is attached at the third brace to allow stable positioning on the testbed.

The hardware described in this paper is a one degree of freedom (DoF) arm exoskeleton, with the joint actuated by a direct-drive brushless direct current (BLDC) motor (Fig. 1). It can generate a torque of 0.5 Nm at stall for 60 s with a current of 10 A without any heating issues. While this peak torque is small, it is strong enough to convey useful torque feedback, and utilizes a very light-weight and low profile motor. If desired, it is straightforward to replace the motor with a more powerful BLDC motor to generate more torque. The mechanical hardware constitutes simple plastic plates and aluminum frames that are easily manufactured in a laboratory or hobby shop environment. In addition to providing details about the exoskeleton structure, we provide source code files for running the motor with full torque at stall conditions as well as software to communicate with a computer and log data from the exoskeleton.

Thus, our system is useful for researchers who are interested in the following tasks:

• Motion training or haptic feedback using an exoskeleton, for example in sports training, rehabilitation, or virtual reality scenarios;
• Laboratory experiments with haptic feedback or wearable robotics; and
• Robotics using high-torque, low-cost hobby motors.

### 2.1 Exoskeleton hardware

The actuator interface is constructed with easily-accessible materials such as 6061 Aluminum and plastic sheets. The actuator interface is a semi-rigid structure made of aluminum frames with plastic covers wrapping around the forearm and upper arm. The motor is mounted to the frame at the hinge. The exoskeleton is tethered to the microcontroller and the power supply. The aluminum frame is on the outside of the wearer’s arm, and wraps half way around the exterior of the arm. The plastic cover wraps around the anterior side of the arm, where muscle volume changes occur during device operation. Foam pads the user inside the plastic covers, and straps hold the wearer inside the exoskeleton.

The exoskeleton is driven by a T-Motor MN7005-KV115 outrunner brushless motor, and its position is sensed by a US Digital S1-5000 optical encoder. The motor is controlled by a Texas Instrument (TI) C2000 TMS320F28069M launchpad as the main processing unit. The microcontroller directly communicates with an attached motor shield, a DRV8305EVM.

Two miniature fans are attached above the motor to circulate the air through it, since it cannot dissipate heat effectively at stall. From testing, we found that the actuator system could handle the heat generated from 10A for more than 30 s (out of a peak current of 15A). The exoskeleton is designed to create up to 0.5 Nm of torque while keeping the weight at 500 g. These specifications are dependent on the motor chosen; with more powerful motors, the maximum torque can be increased.

### 2.2 Software architecture

**Fig. 2:**
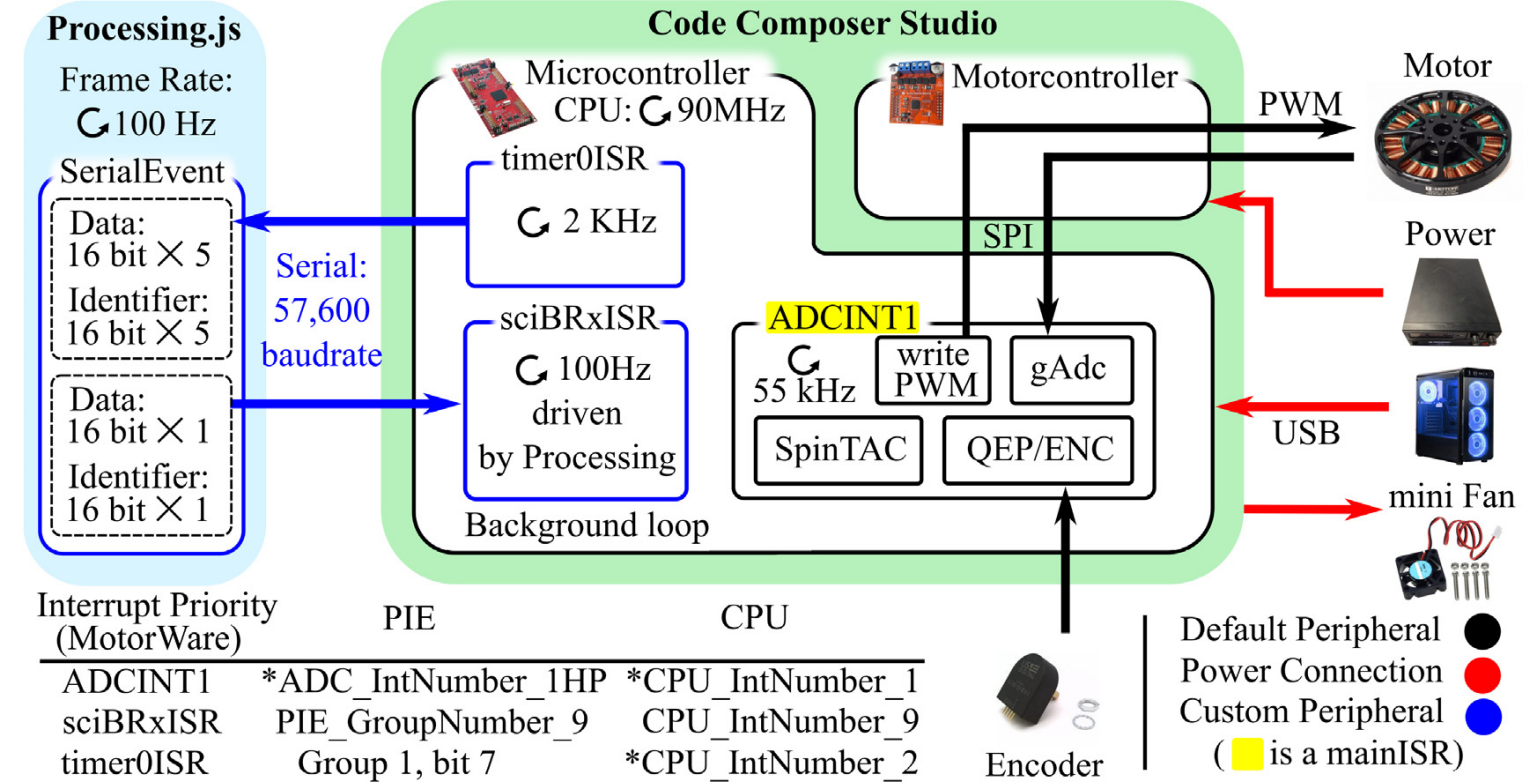
System overview and interrupt routines. Key components with the corresponding peripherals are addressed. (* marked priorities have to be set manually by users).

The embedded software platform is Code Composer Studio (CCS) with a Texas Instrument (TI) C2000 TMS320F28069M as the main processing unit. The microcontroller directly communicates with the attached motor shield, a DRV8305EVM, via Texas Instrument’s MotorWare™ Library (Fig. 2). The TI MotorWare library has been used by several groups for motor control [13], [14] but the details of many elements needed for its effective use have not been provided previously. The MotorWare library employs Field Oriented Control (FOC, equivalent to Vector Control), where the pulse-width modulation (PWM) waveforms are generated based on the relative position between the stator and rotor in the motor. FOC is suitable for controlling Permanent Magnet Synchronous Motors (PMSM) and Brushless DC motors (BLDC).

Our code is an encapsulated library that includes serial communication, timer interrupt, and motor-related functionalities. We use the library in conjunction with TI’s tutorial project files ([15], which provides several “labs” demonstrating the MotorWare code). In our code, we estimate the mechanical angle through a predefined index pin, which allows us to use optical encoder as an absolute encoder while also providing an electrical angle. Bi-directional communication is achieved through a timer interrupt and serial interrupt module. Instead of using the provided position control project (lab13b) where a single bandwidth is used as a tuning parameter, we use a proportional-derivative (PD) controller because it allows more control. It is also more intuitive to tune the PD controller, as each of the gains corresponds to the position and speed error, respectively.

### 2.3 Initial position detection

Estimating an electrical angle is necessary for accurate torque production in vector control. The measured or calculated electrical angle is transformed into three PWM phasors for running the motor, through the Park Transformation and Space Vector Module. The electrical angle is determined based on the position relative to the North and South poles on the rotor and can be measured by a Hall effect sensor. Additionally, for a motor to be used as a direct-drive actuator, a measurement of the mechanical angle (i.e., the exoskeleton joint angle) is necessary. A common approach to obtain both types of angle information is to use both an absolute encoder and a Hall effect sensor. Depending on the choice of an incremental or absolute encoder, the mechanical angle could be relative to the encoder power-up position or the absolute rotation angle, respectively.

Alternatively, TI MotorWare provides three methods for angle estimation: sensorless, hall sensor, and only encoder. The first two cases rely on an internal estimator. In the estimator, the motor model is reconstructed based on the back-electromagnetic field (back-EMF) readings and predefined motor specifications. However, usable back-EMF readings are only available under sufficient speed, and the initial angle estimation becomes challenging during the initial start-up where the back-EMF is weak. Therefore, those methods are not appropriate for a manipulator operating at slow speeds or stall.

The third method implements a current injection technique. The technique is valid in vector control, where a three-phase PWM waveform (a–b–c axes) is transformed from d–q variables. MotorWare uses the phase magnitude invariance method (Chapters 3 and 5 in [16]) for the conversion. The current applied on the quadrature axis (q-axis) is proportional to the generated torque and spatially perpendicular to the main flux direction (d-axis). The injected current on the d-axis should not affect the torque intensity nor causing motor rotation, so long as the estimated d and q axes correctly line up with the actual axes. Therefore, applying current is used as a prerequisite step before running the motor, and it aligns the rotor to the stator position. It also measures the coil resistance for accurate motor control. However, it is not ideal when the load is too heavy to move. In a direct-drive application, or an application with a low-backlash gearbox connected to a load, the motor is not able to move sufficiently with this technique to align the rotor and stator.

Instead, we estimate the mechanical angle via the index-pin, where the initial angle for vector control is measured with an optical encoder and its predefined index pin. By calculating the mechanical angle based on the pre-memorized position through the index pin, we use an incremental encoder like an absolute encoder, and we re-calculate the electrical angle based on the mechanical angle (Fig. 9). As this approach disables the existing initial current injection, which measures the coil resistance, we use runRsOnLine() from [15] to update the coil resistance periodically. This also mitigates the coil resistance drop during the temperature change from stall operation, enabling accurate motor control.

## 3 Design files

CAD models for 3D printing, drawings for aluminium frame fabrication, and CCS scripts for motor control are provided.

**Table:** 

Design filename	File type	Open source license	Location of the file
CAD_Files.zip	3D print files	CC BY-NC-SA 4.0	Mendeley Dataset
CCS_codes.zip	CCS scripts	BSD 3-Clause License	Mendeley Dataset
Processing_codes.zip	Processing scripts	MIT License	Mendeley Dataset
frame_drawings.pdf	pdf Schematic	CC BY-NC-SA 4.0	Mendeley Dataset

## 4 Bill of materials

Table 1 shows a list of components with their labels. Actuator modules are grouped as A; the hardware structure for the exoskeleton is presented in group H; software groups are in S.

**Fig. 3:**
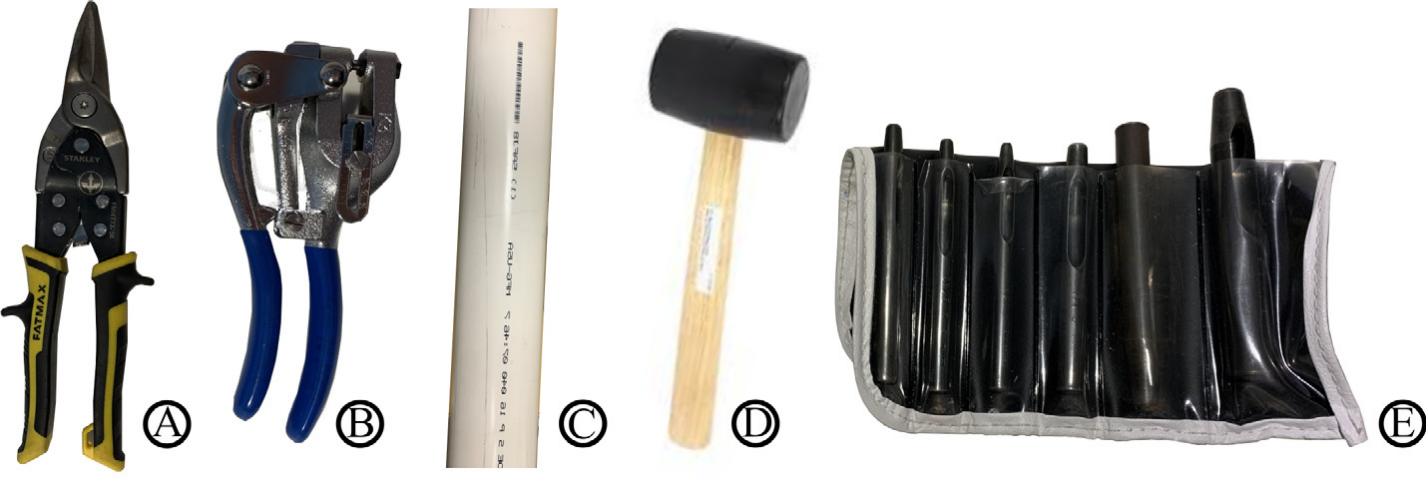
Primary hand tools for fabricating the frame. These include:  FMHT73756 tin snips, ($16–18) – used to cut H1, a long thin beam, into shorter pieces;  Metal Hand Punch ($25–39) – used to punch holes in beams;  PVC Pipe ($3–10) and  Rubber Mallet ($2–10) – used to bend the aluminum braces into the curved arm shape; and  Hollow Punch Tool ($10–20) – creating mounting holes in fabric. Additional required equipment not shown above are: screw drivers, a sewing machine, a soldering iron, a table mount, and a power supply (or a battery).

In addition to the above materials, several hand tools are needed to complete the build process. These are shown in Fig. 3.

## 5 Build instructions

**Fig. 4:**
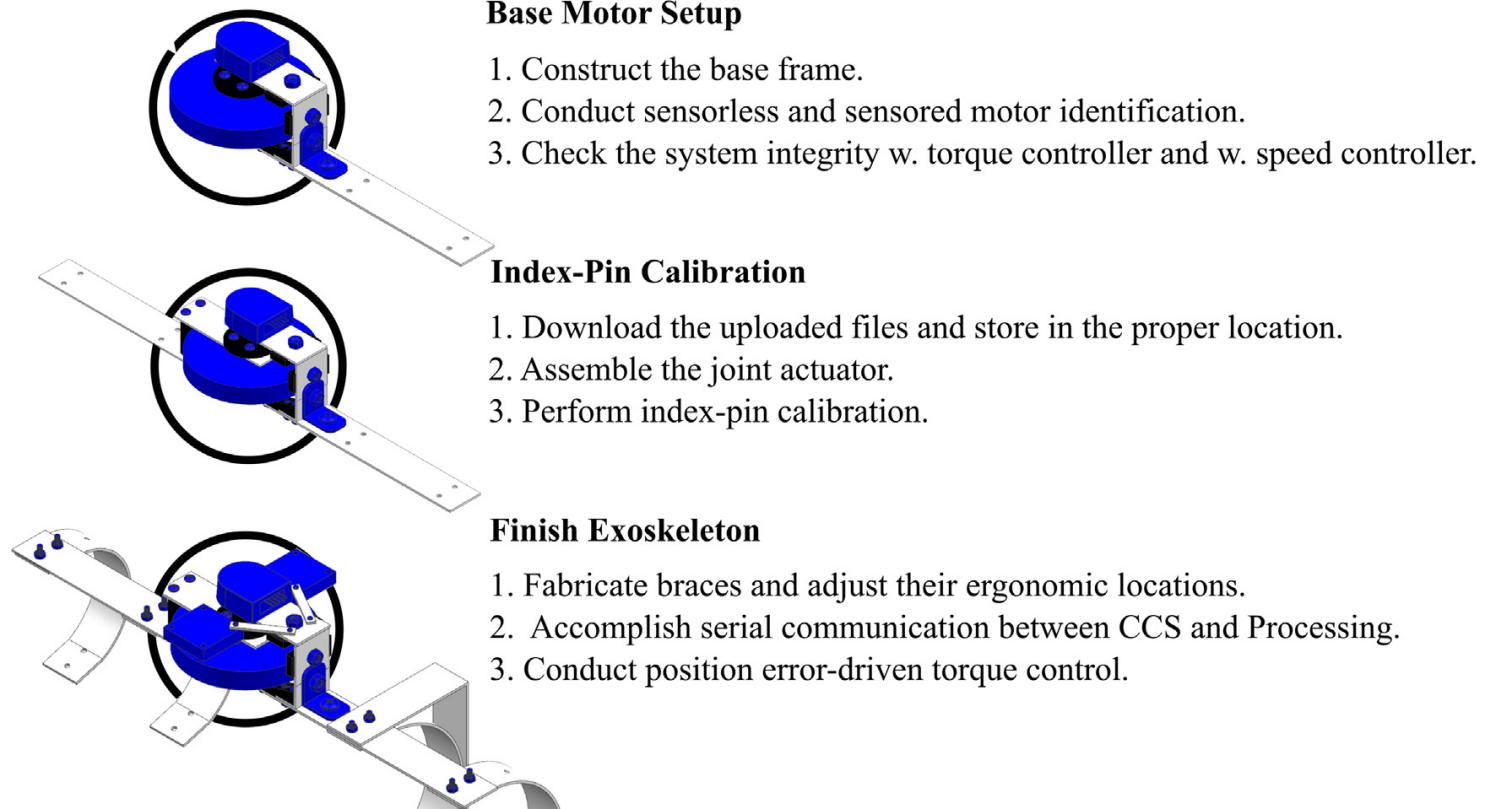
Overview of the assembly process, showing the three stages of assembly discussed in this paper.

**Fig. 5:**
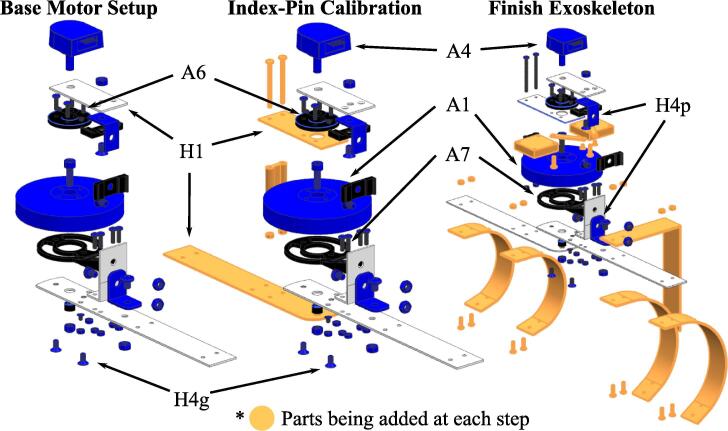
Overview of the mechanical assembly procedure in each stage.

The assembly process involves three primary stages, as seen in Fig. 4: Base Motor Setup, Index-Pin Calibration, and Final Exoskeleton Assembly. Each step includes both hardware and software steps. An overview of the hardware assembled during each step is in Fig. 5. The scope of contents addressed in this paper is more focused on the engineering side based on the existing manuals. Therefore, technical basics provided from [15], such as importing the MotorWare library or checking the target configuration, are not discussed.

### 5.1 Base motor setup

#### 5.1.1 Constructing base frame

**Fig. 6:**
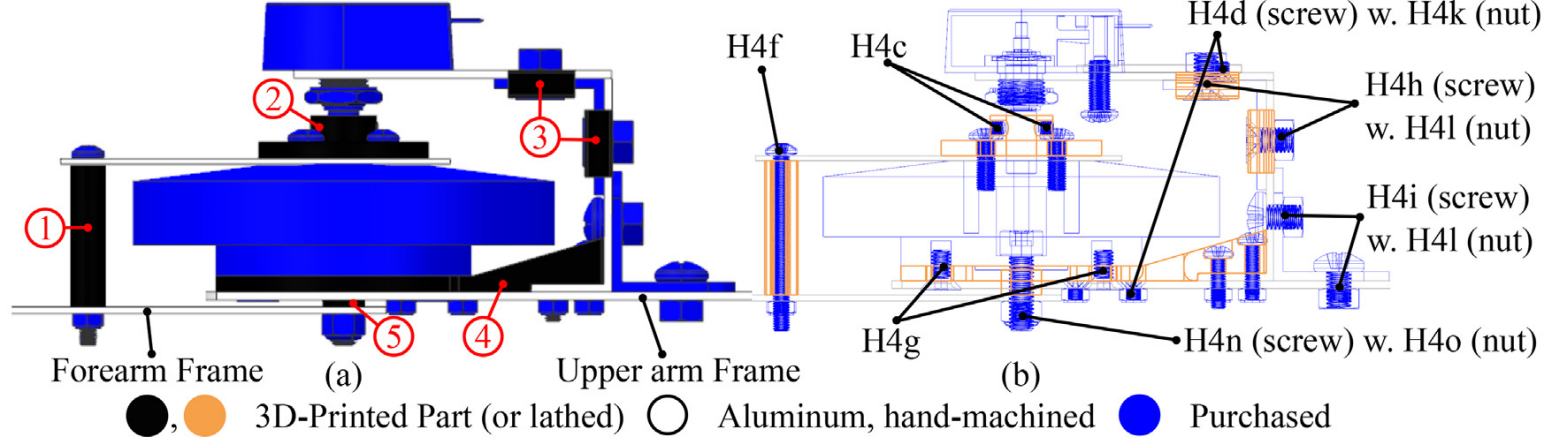
(a), CAD Models with colors indicating the primary method for fabrication. Detailed information about the CAD files (.prt) are ①: forearm_screw_cover (A6a), ②: ENCDAdapter (A6b), ③: dustCover_Top (A6c), ④: EncoderFrame_Bottom (A6d), and ⑤: M4Spacer (A6e). (b), Cross-sectional view with the fasteners indicated. The H4d screws securing the ④ to the Upper arm Frame should be countersunk in the direction from the ④ toward the Upper arm Frame. Similarly, H4g screws assembling Upper arm Frame, ④, and A1 should be countersunk in the direction from the frame to the motor. The holes for setscrews (H4c) of ② should be threaded after being printed or manufactured. Screws not specifically indicated are H4e, M3 × 10 mm; nuts not specifically indicated are H4k, M3.

**Fig. 7:**
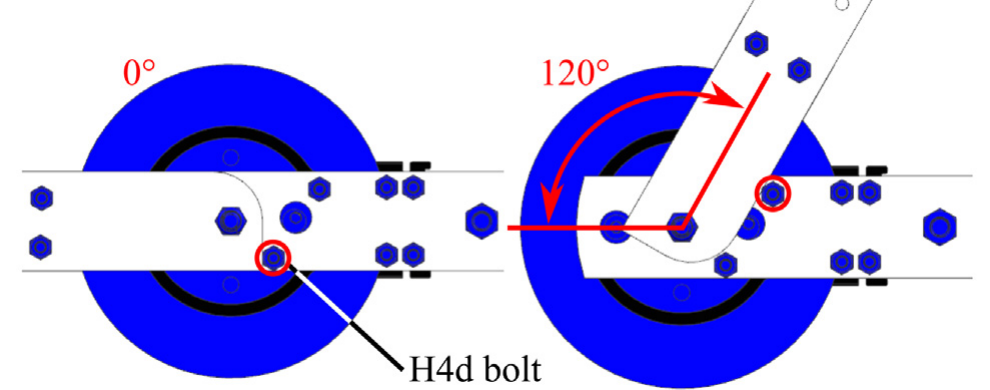
Mechanical hard stops limit the arm movement from 0 to 120 °C. These mitigate any mechanical risks from accidentally overpowering the motor.

The first manufacturing steps are to attach the motor and encoder to the base frame and ensure they are functioning correctly. To complete the base frame, four CAD files (②, ③×2, and ④ in Fig. 6) should be built. Most pieces of the frame are thin aluminum sheet metal (H1), so simple hand tools suffice for most of the manufacturing steps. The fabrication process is easily performed by printing out the downloaded CAD drawings (one to one scale in A3 paper) and pasting them to the Aluminum sheet, which provides a template for cutting or drilling the metal sheet ( and  in Fig. 3). When fabricating sheet metal parts, be careful to keep metal dust away from the motor, as it can get stuck between the rotor and stator and cause it to jam. ② in Fig. 6 is the most critical part in the fabrication process as the motor control relies on the encoder readings. The best way to manufacture ② in Fig. 6 is to use a lathe, although high-resolution 3D-printing works as well. Whether the encoder shaft functions properly or not can be tested using MotorWare lab12b, sensored speed control. Two holes require tapping, indicated by red circles in Fig. 7. These are travel limits for the exoskeleton to prevent over-rotation. When the motor is securely mounted on the upper arm frame through ④ and aligned with the encoder, the motor is ready to be tested.

#### 5.1.2 System integration test

**Fig. 8:**
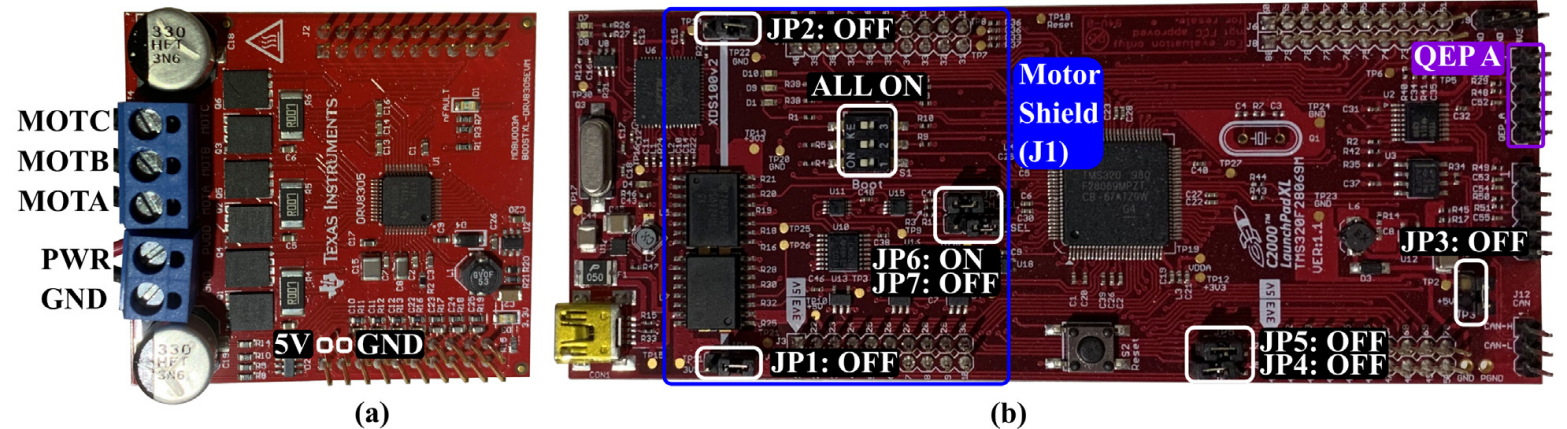
(a) Wiring for the DRV8305EVM motor controller shield and (b) Settings for the C2000 board [17]. In (a), pin 21 and 22 are used for running the Miniature 5 V Cooling fan. In (b), **White** indicates the jumper and switch settings;  is where the motor shield should go;  is where the encoder should be attached.

Following the mechanical assembly of the motor, encoder, and base frame, the hardware and software functionality should be verified. The primary focus of this part is to check if the hardware and original MotorWare projects function appropriately before applying the custom code provided with this paper. First, the jumpers on the C2000 board must be set up correctly, as shown in Fig. 8(b). The motor controller should be attached to the C2000 board, and the motor wires and encoder connected, as in Fig. 8(a).

**Fig. 9:**
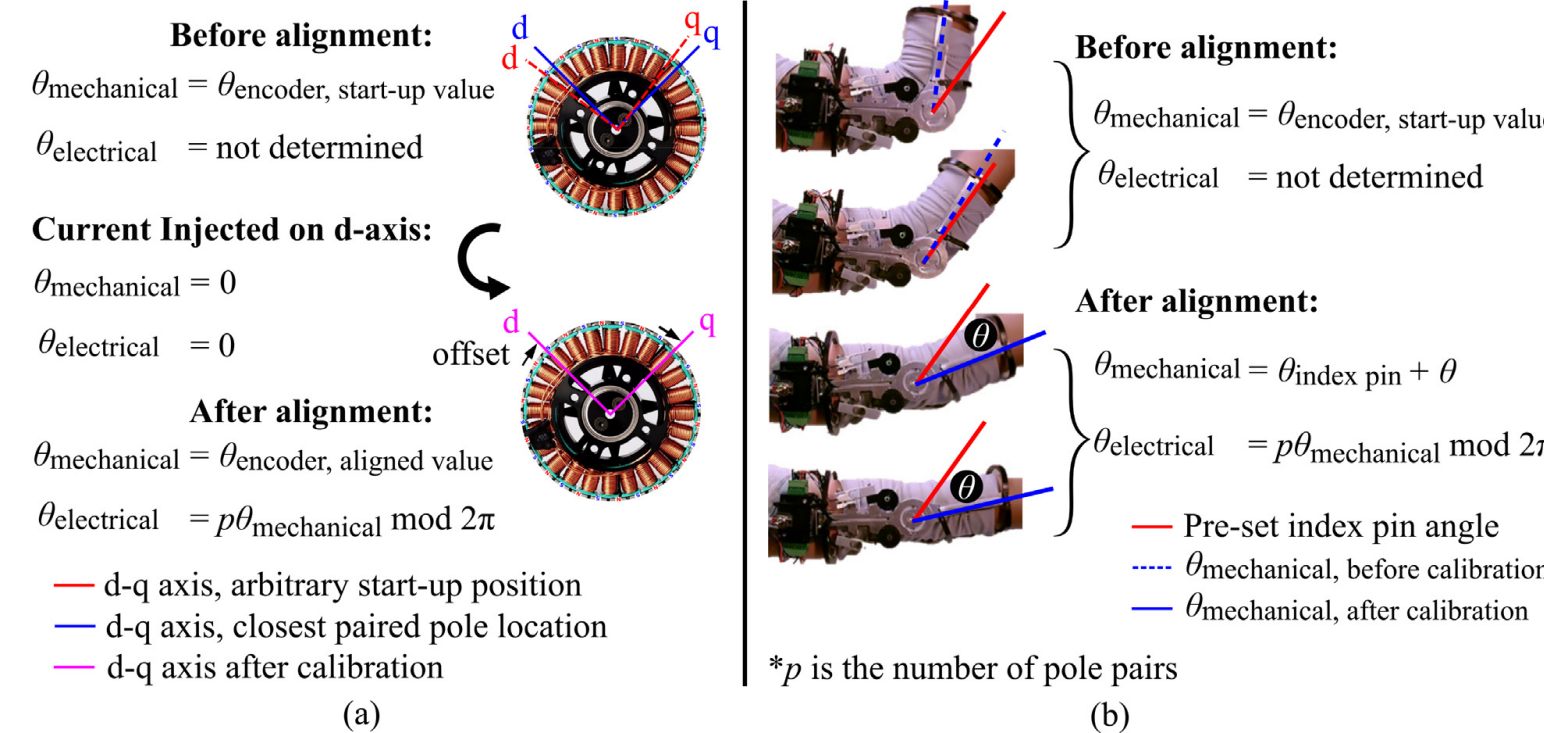
(a), Angle estimation process with TI’s sensored control. The code is programmed to initialize both mechanical and electrical angle to zero as the current is injected. (b), Angle estimation through a predefined index pin, as is done in our system. Current injection is a very useful approach, yet is not feasible for a direct-drive wearable device.

Two steps must be completed before running the motor: (1) figuring out the correct combination of motor windings, and (2) identifying the sensorless motor parameters, such as the winding’s inductance, resistance, and magnetic flux. Both steps are performed simultaneously through TI’s sensorless parameter identification project (lab2a or lab2c, provided by MotorWare [15]). Both projects yield the user_j1.h file for sensorless control, which contains the required parameters; the projects function the same way, but lab2c is for low inductance motors. With a BLDC motor with many pole pairs (i.e., 24N28P), potentially lab2a yields too low of an inductance reading (<50 μH) which will possibly result in an error during other projects. In this case, lab2c should be used. Aside from lab2a and lab2c, the other projects in MotorWare require the identified motor parameters and so cannot be run until the motor parameters are determined. Accordingly, lab2a or lab2c must be used to figure out the correct mapping between motor windings (U, V, and W) and the motor controller’s PWM channels (MOTA, MOTB, and MOTC in Fig. 8(a)). An appropriate winding combination should lead to finishing the parameter identification project (lab2a or lab2c) without any pause in the motor’s rotation, vibration, or unusual behavior (i.e., rotating backward).

MotorWare projects share two versions of motor parameter files; one for the sensorless control (FOC) and another for the sensored (InstaSPIN). Both motor parameter files are named user_j1.h but stored in different paths. Once the sensorless user_j1.h is obtained through lab2a or lab2c, the sensored motor parameters, like motor inertia and friction, should also be measured in lab12a. Sensorless projects utilize the FAST estimator, which runs the motor model internally based on the back-EMF readings. In comparison, sensored projects (e.g. lab12a) use encoder readings. Thus, those two types of projects share different motor parameter files.

To run lab12a, USER_MOTOR_MAX_SPEED_KRPM in user_j1.h has to be set to be 1. While running lab12a, misalignment of the encoder shaft could lead to skipping the index reading, resulting in a malfunction of the motor. The connection between the encoder and motor can be adjusted if this occurs. We use an encoder with a built-in shaft (A4) because in prototype development we found that it was extremely difficult to align a shaft-less encoder with the motor properly.

Upon both the sensorless user_j1.h and sensored user_j1.h being ready, simple motor control can be tested by running the torque controller (lab4) and speed controller (lab12b) that come with MotorWare. A successful run on project lab4 ensures that the sensorless parameters are identified appropriately, and running the speed controller (lab12b) verifies the encoder setup and other aspects of the hardware system.

Following is a summary of the software steps to this point:

• Install MotorWare (S1).
• Prepare the C2000 microprocessor with the proper jumper configuration (Fig. 8).
• Perform Motor Identification (lab2a or lab2c and lab12a) to create both sensorless and sensored versions of user_j1.h.
• Perform torque control (lab4) and speed control (lab12b) to check the system functionality.

### 5.2 Index-pin calibration

In this stage of the build process, we install the custom microcontroller code provided with the paper, as well as set up the encoder and align its index location within an appropriate rotation range of the motor. It is necessary to calibrate the index pin before finalizing the whole assembly because the inertia of the exoskeleton hinders the motor alignment if done at the end.

This section involves changing the peripherals’ priorities. When modifying the peripherals’ priorities all the projects linked to the source file should be updated simultaneously. Thus, after this step, all other projects in MotorWare will not compile.

#### 5.2.1 Custom microcontroller code setup

Following the basic motor setup and testing in the Base Motor Setup stage, this section addresses how to set up the custom microcontroller code provided with this paper. The microcontroller source code files provided with this paper (S1) are based on lab12b and thus share all the embedded features with that project. Linking scripts (S1b and S1d) to the lab12b workspace (WS) and replacing one header file (S1c) enables us to access the key features in MotorWare. All files (S1) should be located on the specific path MW_INSTALL_DIR⧹sw⧹solutions⧹instaspin_motion⧹src. The default path of MotorWare is \path{C:\ti\motorware\motorware_1_01_00_18\} and its relative path is defined by Eclipse as \path{MW_INSTALL_DIR}. One essential script that is not included on the list but still required to be linked to the WS is the sci.c file. The file can be found in MW_INSTALL_DIR⧹sw⧹drivers⧹sci⧹src⧹32b⧹f28x⧹f2806x. To link the script to an existing WS, use “Add files” from the project context menu of lab12b, which is accessible by right-clicking the project name on the Project Explorer. Then locate where the downloaded the scripts (S1b and S1d) are. Create links to the files relative to PROJECT_LOC. Detailed steps to link files to the current WS is explained in Section 6.7 in [18]. After linking the custom code, remove the original lab12b.c link file as the .obj file is one-to-one mapped with the .c file.

In the custom code, we introduce several interrupt routines to the system. Since the main Interrupt Service Routine (ISR) of the MotorWare is directly related to the PWM generation, ordering the priorities of these interrupts is critically important for the system’s proper functioning. Fig. 2 shows two different priorities, CPU and PIE. Both are part of the interrupt management levels of Digital Signal Processing. If an interrupt event takes place and interrupt flag bit is on, the interrupt request is delivered to the PIE controller. The request is then managed at the CPU level (Chapter 1.7 in [19]). Among priorities illustrated in Fig. 2, the PIE priority of the timer0ISR() is the default setting and does not need any modification. Both PIE and CPU settings of the sciBRxISR() are declared as the uploaded HardwareX_comm_N_timer.c is linked to the WS. The CPU priority of the timer0ISR() and both priority settings of ADCINT1() need to be manually changed. Two relevant files, hal.h and hal.c of the Hardware Abstract Layer, address the general peripherals’ priorities and initialization [18] and must be edited as follows:

In hal.h:

**Table:** 

at “the globals”,	add: extern interrupt void sciBRxISR(void);
	add: extern interrupt void timer0ISR(void);
at HAL_acqAdcInt(),	change the parameter of PIE_clearInt() from
	PIE_GroupNumber_10 to PIE_GroupNumber_1
at HAL_initIntVectorTable(),	change the PIE priority of pie->ADCINT1 = &mainISR; from
	pie->ADCINT1 to pie->ADCINT1_HP
	add: pie->SCIRXINTB = &sciBRxISR;
	add: pie->TINT0 = &timer0ISR;

Similarly, in hal.c:

**Table:** 

at HAL_enableAdcInts(),	change the parameter of PIE_enableAdcInt() from
	ADC_IntNumber_1 to ADC_IntNumber_1HP
	change the parameter of CPU_enableInt() from
	CPU_IntNumber_10 to CPU_IntNumber_1
at HAL_enableTimer0Int(),	change the parameter of CPU_enableInt() from
	CPU_IntNumber_1 to CPU_IntNumber_2;

#### 5.2.2 Processing code setup and serial communication with CCS

Following the setup of the initial microcontroller code, we need to prepare the computer code. Download and prepare the Processing script and select the appropriate COM port for serial communication. If CCS has trouble connecting to the board, make sure that the TI’s COM Port has the virtual terminal option on, in the Device Manager. The port number set should be an auxiliary port for TI. Two supplementary files, the communication states file (receive_Stat.java - S2b) and the pre-programmed trajectory (traj_1.txt - S2c), have to be in the same folder with the Processing_HardwareX.pde.

#### 5.2.3 Assemble the joint actuator

We next assemble the forearm side of the exoskeleton frame. The forearm assembly is attached to the Base Motor Setup (Fig. 5, Fig. 6) with the 3D-printed spacer (⑤ in Fig. 6) in between the aluminum frames. An M4 screw (H4n) passes through the motor (A1), motorFrameAdapter ④, and frameSpacing ⑤ , and is secured with a lock nut (H4o). The most important part of the assembly is that the index pin should be in the range of motion. To assure this, the reading values for the index pin should be monitored during the assembly process via the Real-Time Watch Window in CCS. The entire operation steps are closely related to TI’s state machines of: Flag_enableSys – system flag to initiate background loop, Flag_Run_Identify – flag to check the motor parameter before Controller API, CtrlState – state for the Controller API, and EstState – state for Estimator API. With the motor and encoder connected, but not yet secured:

1. **CCS**: At the top of the haptic_joint.c, locate where all the defines are declared. Uncomment AngleResetViaIndxPin and comment AngleResetViaIndxPin_Dbug out.
2. **CCS**: Build the project: Debug project, Enable Silicon Real-time mode, Enable continuous refresh, and Resume.
3. **Processing**: Execute the Processing_HardwareX.pde by pressing Run button. This initiates interrupt events for sciBRXISR() and timer0ISR() on the microcontroller. Check if Flag_enableSys is 1, and Flag_Run_Identify is 0 in the Real-Time Watch Window, Expressions tab.
4. **CCS**: Create a variable mtrPosInitiated in bool format in the Real-Time Watch Window, Expressions tab.
5. Try rotate the motor from the initial arm angle to the desired maximum angle, and see if the mtrPosInitiated variable changes to 1.

Repeat step 5 until the index-pin is detected within the range of motion. The index pin on the encoder should be verified to be located within the arm’s traveling range before finalizing the forearm frame’s location relative to the motor and proceeding to subsequent steps (Fig. 9). Secure the encoder to the motor with setscrews (H4c) in part ② after the index pulse is observed and terminate the system by pressing the space bar key.

#### 5.2.4 Perform index-pin calibration

The following are the steps to calibrate the index pin (Fig. 9) after the hardware assembly. As the encoder reading determines the electrical angle, running the motor without calibration might lead to unexpected motor operation.

1. **CCS**: At the top of the haptic_joint.c, locate where all the defines are declared. Uncomment AngleResetViaIndxPin and comment AngleResetViaIndxPin_Dbug out.
2. **CCS**: Build the project: Debug project, Enable Silicon Real-time mode, Enable continuous refresh, and Resume.
3. **CCS**: Create variables, debug_IndxCnt_1 and debug_zeroOffset in the Real-Time Watch Window, Expressions tab.
4. **CCS**: Stop the code and terminate.
5. **CCS**: Uncomment both define:AngleResetViaIndxPin and AngleResetViaIndxPin_Dbug.
6. Make the exoskeleton fully extended (straightened) before initializing the code. Also, make sure the motor is flat on the ground to mitigate possible tilting.
7. **CCS**: Build the project: Debug project, Enable Silicon Real-time mode, Enable continuous refresh, and Resume.
8. **Processing**: Execute the Processing_HardwareX.pde by pressing Run button. This initiates interrupt events for sciBRXISR() and timer0ISR() on the microcontroller. Check if both Flag_enableSys and Flag_Run_Identify are 1 in the Real-Time Watch Window, Expressions tab.
9. **CCS**: As the current is injected, motor aligns itself to the closest paired pole location as in Fig. 9(a). The current injection process also can be monitored through the power supply. Occasionally, the motor rotates even after the alignment. In this case, restart from step 1.
10. **CCS**: Record value in debug_zeroOffset, save it for later use. This is the offset between the desired initial position and the closest paired pole location.
11. Swing the exoskeleton to the maximum range of motion.
12. **CCS**: Record the debug_IndxCnt_1 value for later use. This shows where the index pin is located.
13. **Processing**: Press a space bar to terminate the system.
14. In the timer0ISR(), haptic_joint.c, replace the default value in ENC_setZeroOffset() with the debug_zeroOffset. Also, replace the default value for IndxOffset with the measured debug_IndxCnt_1.
15. **CCS**: This step is to verify the current setting works. Rotate the motor to an arbitrary position. Uncomment AngleResetViaIndxPin and comment AngleResetViaIndxPin_Dbug out. Repeat the Step 6.
16. Execute the Processing_HardwareX.pde by pressing Run button. Check if Flag_enableSys is 1 and Flag_Run_Identify is 0 in the Real-Time Watch Window, Expressions tab.
17. **CCS**: Add a variable mtrPosMrev_iq in the Q-Value(24) format in Real-Time Watch Window. The reading from the variable is an arbitrary reading recorded when powering up the encoder.
18. **CCS**: Move the motor until mtrPosInitiated changes from 0 to 1. When the index-pin is detected, the value of mtrPosMrev_iq should be updated to the calibrated position.
19. **Processing**: Press a space bar to terminate the system.

Whenever the actuator module requires re-assembly, the index pin should be re-calibrated. However, once we set the index pin value, the value should not be changed unless the encoder shaft slips or is detached. In summary,

• Download the code files associated with this paper (S1) and extract them into the appropriate path (Section 5.2.1). Note that main.h replaces the original file and the other files are simply pasted there.
• Link files, S1b and S1d, to the lab12b WS (including sci.c).
• The existing priority settings should be adjusted in hal.c and hal.h.
• Set up Processing (S2) and test the serial communication.
• Assemble the encoder and the forearm frame, making sure the index pin location is in the range of the exoskeleton’s rotation.
• Calibrate the index pin following the procedure in Section 5.2.4.

### 5.3 Finish exoskeleton

#### 5.3.1 Fabricate braces and adjust their ergonomic locations

In this stage, we finish assembling the aluminum braces, taking into consideration the ergonomic requirements of the arm. Just like the aluminum manufacturing process for the Base Motor Setup stage, please refer to the CAD drawings (frame_drawings.pdf). Fig. 5 shows the new components added in this stage.

**Fig. 10:**
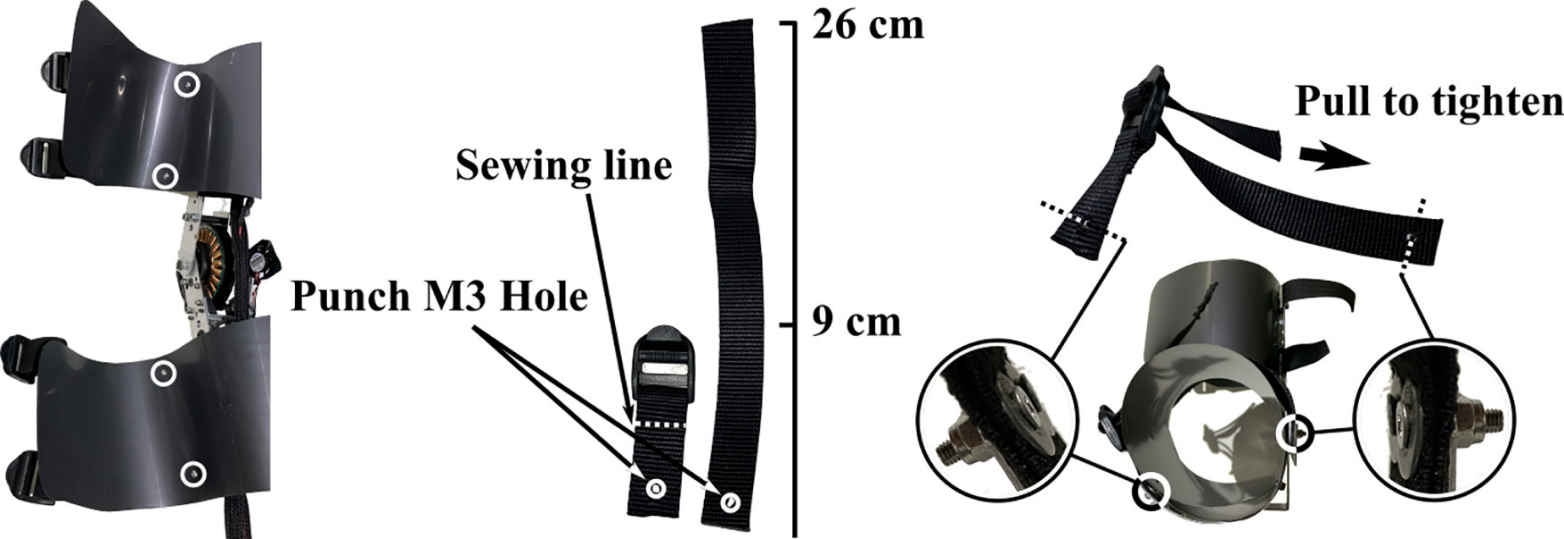
Left: four highlighted screw (H4j) points to secure the Nylon sheet to the frame. Middle: manufacturing guideline for straps (H4a) and buckles (H4b) as where to sew, punch, and screw. Right: mounting process to frames. From the medial to the lateral side, secure the screws in the order of: screw (H4e); washer (H4m); straps (H4a); lock nut (H4k).

To assemble the rest of the exoskeleton, first bend the aluminum strips using the PVC pipe  and hammer  (see Fig. 3). Once the aluminum frames and braces are assembled, attach the Nylon sheet (H3) to the frames with screws (H4j) (Fig. 10). The Nylon sheet is cut to 12 cm × 38 cm for the forearm piece, and 11 cm × 48 cm for the upper arm piece. Foam (H2) i5 also cut to be similar sizes. It could be attached to the Nylon sheet with hot glue, although in our instructions we leave them as separate pieces. The foam provides additional comfort to the wearer and helps the exoskeleton conform to various arm geometries. Next, attach the miniature fans (A5) which are necessary to allow the motor to dissipate heat during stall with screws (H4d and H4e). Finally, sew and punch ( in Fig. 3) the straps (H4a and H4b), and attach them to the braces with screws (H4e in Table 1).

**Table 1:** Bill of materials. “Desig.” = Designator; “Qty.” = Quantity; T = thickness; W = width; L = length.

Desig.	Component	Qty.	Cost pe unit (USD)	Total cost (USD)	Source of materials
					
A1	MN7005 KV115	1	249.9	249.9	T-Motor
A2	LAUNCHXL-28069M	1	24.99	24.99	TI
A3	BOOSTXL-DRV8305EVM	1	93.22	93.22	Digi-key
A4	S1 Optical Shaft Encoder	1	89.80	89.80	US digital
A5	Miniature 5 V Cooling fan	2	3.50	7.00	Adafruit
A6	A6a. forearmScrewHousing	1	–	–	3D print
	A6b. encoderMotorAdapter	1	–	–	3D print
	A6c. encoderAdjustment	1	–	–	3D print
	A6d. encoderFrameAdapter	1	–	–	3D print
	A6e. frameSpacing	1	–	–	3D print
H1	6061 Aluminum, 1/16”T, 1”W	1	4.74	4.74	McMaster
H2	Foam Sheet, 1/4”T, 18” by 20”	1	16.54	16.54	McMaster
H3	Nylon Sheet, 6”W, 0.015”T	1	12.80	12.80	McMaster
H4	H4a. Webbing Strap, W: 1” L: 1’	1	0.91	0.91	McMaster
	H4b. Sew-On Buckles for 1” Webbing	4	0.28	1.12	McMaster
	H4c. M3-0.5 × 3 mm Set Screw	2	0.05	0.10	McMaster
	H4d. M3-0.5 × 4 mm Flat Head	4	0.15	0.60	McMaster
	H4e. M3-0.5 × 10 mm Flat Head	18	0.05	0.90	McMaster
	H4f. M3-0.5 × 39 mm Flat Head	2	0.10	0.20	McMaster
	H4g. M4-0.7 × 8 mm Flat Head	2	0.05	0.10	McMaster
	H4h. M5-0.8 × 10 mm Flat Head	2	0.07	0.14	McMaster
	H4i. M5-0.8 × 6 mm Flat Head	2	0.32	0.64	McMaster
	H4j. M3-0.5 × 6 mm Flat Head	4	0.05	0.20	McMaster
	H4k. M3-0.5 Nylon-Insert Locknuts	24	0.04	0.80	McMaster
	H4l. M5-0.8 Nylon-Insert Locknuts	4	0.01	0.04	McMaster
	H4m. M3 3.2 mm ID 6 mm OD Washer	8	0.03	0.24	McMaster
	H4n. M4-0.7 × 15 mm Socket Head	1	1.56	1.56	McMaster
	H4o. M4-0.7 Nylon-Insert Locknuts	1	0.12	0.12	McMaster
	H4p. L-Bracket, 7/8” × 7/8” × 5/8”	2	0.43	0.86	McMaster
S1	S1a. HardwareX_comm_N_timer.h	–	–	–	CCS script
	S1b. HardwareX_comm_N_timer.c	–	–	–	CCS script
	S1c. main.h	–	–	–	CCS script
	S1d. HardwareX_haptic_joint.c	–	–	–	CCS script
S2	S2a. Processing_HardwareX.pde	–	–	–	Opensource
	S2b. receive_Stat.java	–	–	–	Opensource
	S2c. traj_1.txt	–	–	–	Opensource

#### 5.3.2 Conduct position error-driven torque control

The exoskeleton provides a torque as a function of the position error:

(1) $$I=P(x-x\_d)-D\overset{˙}{x}$$

(2) τ=K_tI

where *I* is the current, *P* is the stiffness gain, *D* is the damping gain, *x* is the mechanical position, x_d is a specified desired position transmitted from Processing, and $\overset{˙}{x}$ is the filtered speed feedback. τ is the torque created by the motor, and K_t is the motor torque constant. In the microcontroller code, *x* and x_d have units of [revolutions], $\overset{˙}{x}$ has units of [krpm], τ has units of [Nm], and *I* has units of [PU] (“per unit”) where the current is divided by a user-defined full-scale current. Our Processing code provides two different modes for position input, such that the controller could be tuned before applying a predefined path. One is direct-position input, where the experimenter adjusts the position input by pressing up/down on the keyboard. This mode is appropriate for tuning the PD controller before applying any motion trajectory. Another mode is the preprogrammed trajectory. The sample external trajectory file, traj_1.txt, is loaded via multi-threading and sent to CCS. The provided sample path is in degree and is updated in Processing at 100 Hz. Users can switch between the two modes by toggling positionInputMode: 0 for direct-input, and 1 for preprogrammed path.

The P and D gains for the torque control are empirically tuned and can be adjusted according to the desired actuator bandwidth. The current feeding into the system can be observed by adding a variable iqReference in the Q-Value(24) format in Real-Time Watch Window. When performing real-time tuning, variables corresponding to the P, D gains should be typed in Real-Time Watch Window: tuning_KP and tuning_KD in the Q-Value(24) format. Start with a very low number (i.e., 0.001) to begin the tuning safely and then increase the value after observing how the exoskeleton responds. Usually, the P gain value should be tuned first to roughly estimate how fast the motor should rotate, followed by adjusting the D gain to alleviate the overshoot. Typically, motors with strong static friction require higher P gains. If the motor is unstable upon powering up (i.e., vibrates), the system requires a higher damping value to be stable. In summary, in this stage the steps are:

• Fabricate the braces and attach them.
• Attach the Nylon sheet (H3).
• Connect the fans.
• Sew and punch () straps, tailor them, and link them to the braces.
• Empirically tune the P and D gains for position-error based torque controller.

### 5.4 Design considerations

In this section, we elaborate on design considerations for the system. Specifically, we provide additional details on designing the human interface, sizing a motor, and improving communication between Processing and the microcontroller. The process outlined in this paper is adaptable to other motors besides the one selected for this project; this section explains how to choose a new motor and use it with the software.

#### 5.4.1 Designing the human interface

The frame design should allow the exoskeleton to be comfortable throughout the arm’s range of motion. Instead of a metal frame completely surrounding the arm, the arm braces on the medial side are made of soft fabric while the bottom half remains made of rigid parts. This enables the braces to deliver the torque from the motor while not impeding the range of motion when the arm is fully flexed. Second, the exoskeleton should adapt to the muscle volumes, as the arm muscles can change drastically during arm movement. This is accomplished by the plastic sheets held in place by elastic straps. Third, the foam on the inside of the exoskeleton conforms to the wearer, as the plastic sheets are cylindrical, and also provide a soft interface. The exoskeleton can be adjusted for different wearers’ geometries by bending the aluminum or altering the foam thickness.

#### 5.4.2 Sizing motors for passive control

**Fig. 11:**
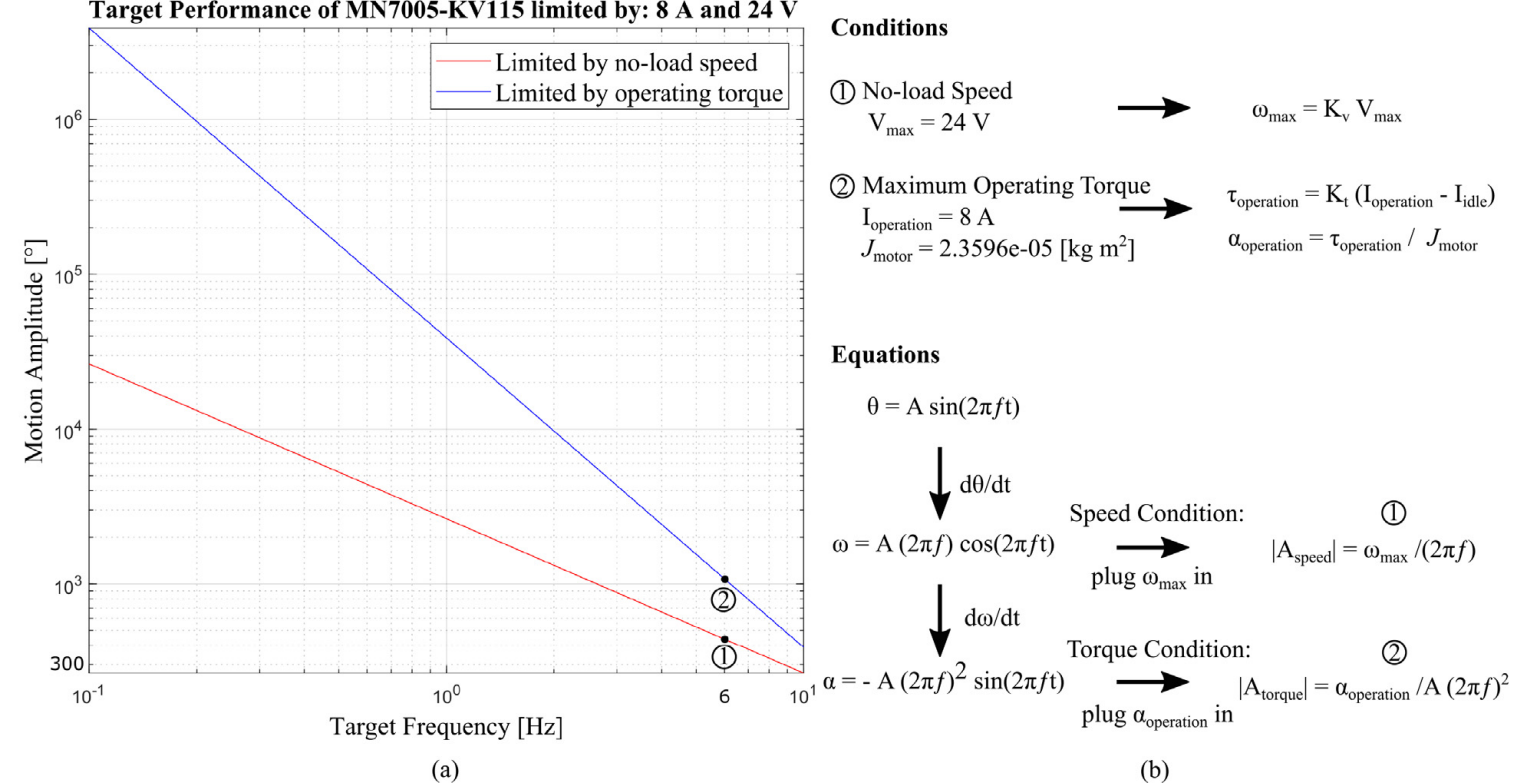
(a), Simulation of the exoskeleton described in this paper. The motion amplitude achievable at different frequencies. Both conditions yield motion amplitudes of more than 300° at 6 Hz, which is larger than our target amplitude of 50°. Note that the peak-to-peak angle of the motion is twice the amplitude. The simulations assume a voltage of 24 V and a maximum operating current of 8 A. (b), Summary of the calculations for how to determine the required motor size. In the last steps for finding |A_speed| and |A_torque|,cos(2πft) or sin(2πft) are set to 1, as that is the maximum value. J_motor is identified from lab2c and converted using an equation (Section 10.5 in [15]). The inertia of the exoskeleton frame is neglected in this example.

Passive control in motion training can be simplified to an actuator’s motion: the actuator applies the desired motion regardless of the wearer’s force feedback. The problem can be easily analyzed if the desired motion is a simplified sinusoidal motion, and we ignore any interaction forces with the wearer. An overview of the calculations needed to select a motor are shown in Fig. 11, and the equations are also as follows:

(3) ω_max=K_vV_max

(4) τ_operation=K_t(I_operation-I_idle)

(5) α_operation=τ_operation/J_motor

(6) θ=Asin(2πft)

(7) ω=A(2πf)cos(2πft)

(8) $$\alpha =A{\left(2\pi f\right)}^{2}\sin(2\pi \mathrm{ft})$$

(9) |A_speed|=ω_max/(2πft)

(10) $$|A\_\mathrm{torque}|=\alpha \_\mathrm{operation}/{\left(2\pi \mathrm{ft}\right)}^{2}$$

where V_max is the maximum voltage; ω_max is the maximum motor speed; K_v is the motor velocity constant; K_t is the motor torque constant; I_operation is the maximum operation current; I_idle is the no-load current; J_motor is the motor rotor inertia; α_operation is the angular acceleration of the motor; τ_operation is the motor torque; θ is simulated motion; *A* is the magnitude of the trigonometric functions; and *f* is the frequency, which ranges from 0.1 to 10 Hz in Fig. 11. The angular speed (Eq. 7) and angular acceleration (Eq. 8) are generated from the simplified motion equation (Eq. 6) and taking derivatives.

The motor performance is evaluated in two aspects: no-load speed and maximum operating torque. The chosen motor’s velocity constant (K_v) determines the no-load speed (Eq. 3), given the maximum voltage. In comparison, the motor torque constant (K_t), motor inertia, and the maximum operating current decide the operating torque condition (Eqs. (4), (5)). The maximum operating current should be smaller than the rated peak current since the joint actuator mostly operates in a stall condition.

To determine the sinusoidal motion amplitude possible at different frequencies, the maximum angular velocity ω_max is substituted into Eq. (7) and the amplitude is solved for, after setting cos(2πft) to 1 since that is the maximum value. This results in Eq. (9), which is plotted as the red line in Fig. 11(a). Similarly, the amplitude limited by the maximum torque is found by substituting a constant α_operating into Eq. (8) and setting sin(2πft) to 1, and this is plotted in Fig. 11(a) as the blue line. The target performance of 50° amplitude for 6 Hz as the maximum human arm motion can be easily achieved with the given motor inertia. However, the inertia of the attached exoskeleton frame is neglected in the simulation.

Some parameters utilized in the calculation are retrieved from the specification sheet [20], which is measured under continuous rotation. Therefore, parameters sensitive to the heat, such as the coil resistance, could be different when it comes to running the motor under stall condition, and some care should be taken when doing calculations.

In general, for direct-drive applications, outrunner BLDC motors are especially suitable because of their high torque-to-weight ratio. When selecting a specific motor, a high pole-pair number leads to a lower cogging effect from the magnets being attracted to the stators.

#### 5.4.3 Improving communication

**Fig. 12:**
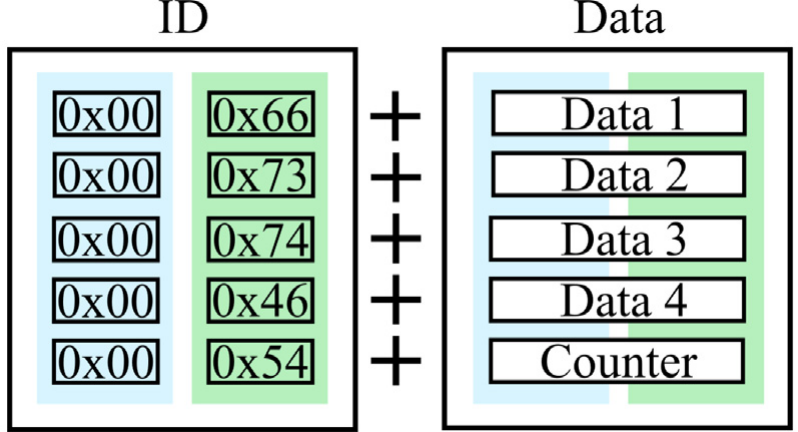
Data Package for logging from CCS. The package is made of 10 bytes of ID and 10 bytes of data (five 16 bit data). Five data IDs, 0x66, 0x73, 0x74, 0x46, and 0x54, are represented as dataID_01, dataID_02, dataID_03, dataID_04, and dataID_Timer in void serialEvent(), Table 2. The IDs are arbitrary chosen and could be changed to different characters. The five variables currently being logged are motor position, filtered torque, current feedback, commanded current, and timer counter.

Table 2 provides an overview of the communication between CCS and Processing. As shown there, the communication process between the microcontroller and Processing involves three interrupt routines, serialEvent() of Processing, and the timer0ISR() and sciBRxISR() of CCS [19]. The current serial communication setting has one stop bit with no parity check. To transmit 20 Bytes of the data package, illustrated in Fig. 12, the number of actual bits delivered via serial frames is 200 bits (Table 2). For the selected baud rate of 57600 (Table 3), which is a bit rate of 1.74E−5, the data package can be sent at around 288 Hz. This is more than twice as fast as the update framerate of Processing_HardwareX.pde, which is 100 Hz.

**Table 2:** Pseudo-code for communication. The ASCII IDs tagged for the current data package are c, 125, 124, and 123 in decimal. Those are arbitrarily chosen and can be modified to be other ASCII codes. Switching to other variables can be done in updateDLogPkg().

**Communication Algorithm**	
serialEvent() in Processing	sciBRxISR() in Code Composer Studio
toggle ← False	toggleCMDIn ← False
command ← desiredInput	dataGet8bit ←getDataNonBlocking()
**if** serial port available **then**	**if** toggleCMDIn == True **then**
incoming8bit ← in *short*	Store: LSB → MSB and Compose
**if** toggle == True **then**	**else**
**switch** state_dataID	**switch** dataGet8bit
**case** == state_Data01	**case** c
Store: LSB → MSB and Compose	toggleCMDIn ← True
**case** == state_Data02	**case** 125
Store: LSB → MSB and Compose	enable: Motor State and timer0ISR
**case** == state_Data03	**case** 124
Store: LSB → MSB and Compose	disable: Motor State and timer0ISR
**case** == state_Data04	**case** 123
Store: LSB → MSB and Compose	initializeExperiment ← True
**case** == state_Timer	**end switch**
Store: LSB → MSB and Compose	**end if**
Transmit: command	timer0ISR() in Code Composer Studio
Update: savingDataArray	flat_updatePkg ← False
**end switch**	HighOrLow ← False
**else**	updateDLogPkg()
**switch** incoming8bit	dataPointer = (uint16*) dataPointerArray [cntr]
**case** == dataID_01	**if** HighOrLow == True **then**
state_dataID ← state_Data01	cntr++
**case** == dataID_02	transmit8bit ← (*dataPointer ≫8) & 0xFF
state_dataID ← state_Data02	**else**
**case** == dataID_03	transmit8bit ← (*dataPointer) & 0xFF
state_dataID ← state_Data03	**end if**
**case** == dataID_04	SCI_putDataNonBlocking(transmit8bit)
state_dataID ← state_Data04	HighOrLow ^= 1
**case** == dataID_Timer	cntr %= size(dataArray)
state_dataID ← state_Counter	**if** cntr == 0 **then**
**default**	flat_updatePkg ← True
toggle ← True	**end if**
LSB ← 0	updateDLogPkg() in Code Composer Studio
MSB ← 0	**if** flat_updatePkg == True **then**
**end switch**	Update: dataArray
**end if**	flat_updatePkg ← False
**end if**	**end if**

**Table 3:** Values for BRR, the 16-bit baud-select register. The default enumeration structure defined in sci.h assumes a serial clock of 15 MHz (low speed peripheral clock, LSPCLK), where setting BRR to 194 leads to a 9600 baud rate. This system described in this paper uses a Baud rate of **57600**. For the detailed table, refer to SPRUH18G [19], Table 13–11.

BRR in decimal	BRR in binary	Baud rate, 15 MHz of LSPCLK	Baud rate, 90 MHz of LSPCLK
194	11000010	9600	**57600**
97	1100001	19200	115200
33	100001	57600	330882
15	1111	115200	703125
9	1001	1125000	1125000

From timer0ISR(), the data array is a pointer array and updated every other timer0ISR() call (Table 2). The bit shifting on CCS side is written as little-endian as C2000 only supports the little-endian format. To implement the data package in both Processing and CCS, we use short in Processing and unsigned int16 in CCS as the data type storing the received data. Note that timer0ISR() triggers updateDLogPkg() with the MotorWare’s default framerate of the timer0, 2 kHz. To synchronize all five data (including the counter in Fig. 12), updateDLogPkg() does not refresh until timer0ISR() finishes sending the whole packet. Ten iterations of data transmission are required for every data logging packet (five 16-bit numbers, transmitted 8 bits at a time at each timer0ISR() interrupt) and so the full data packet updates at 200 Hz. Within Processing, the function void keyPressed() enables users to use keyboard as an input source. Currently, the coded keys are: 1) arrow up/down, 2) Enter, and 3) space bar. These can be switched to be different keyboard inputs.

## 6 Operation instructions

### 6.1 Exoskeleton donning instructions

**Fig. 13:**
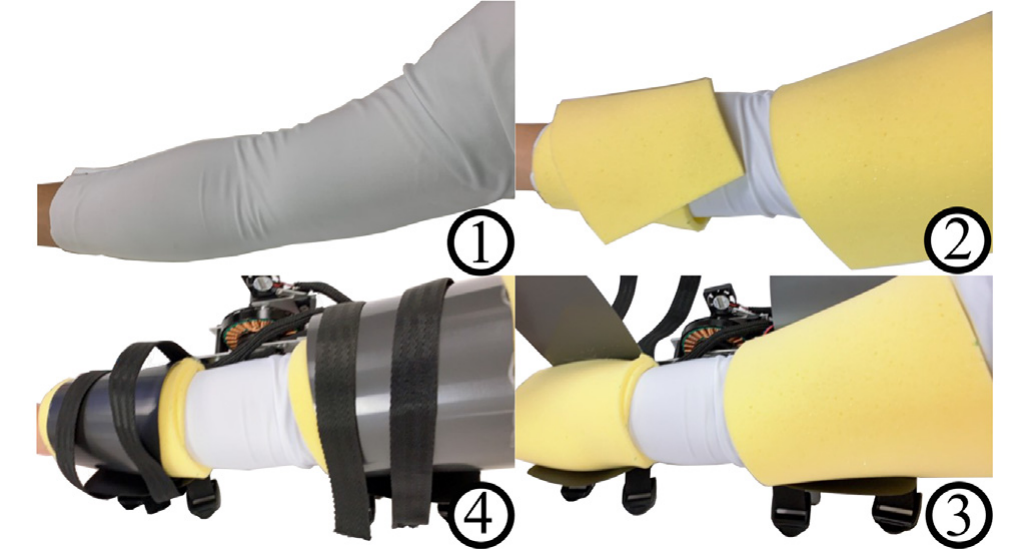
Instructions for donning the exoskeleton, in order counter-clockwise. First, a sleeve is put on the wearer, followed by wrapping the arm in foam. Next, the exoskeleton is wrapped around the foam, and the straps tightened.

The donning process is shown in Fig. 13. First, wear the foam to mitigate any possible discomfort between the plastic cover and skin. The foam is simply wrapped around the arm such that different sizes of arm volumes can be accommodated easily. Second, align the elbow joint with the motor. Third, make sure the plastic cover overlaps internally and tighten the straps. The plastic material cover should slide in easily and secure the foam to the arm.

### 6.2 Activation instructions

Initialization steps are conducted in both CCS as a debugging tool and Processing as a User Interface. Steps for enabling the ISRs and activating the motor are separated, as the index pin reset module instantly updates the current arm position relative to the index pin position. This causes an abrupt torque surge if it occurs when the motor is enabled. The steps to use the device as follows:

1. **CCS**: At the top of the haptic_joint.c, locate where all the defines are declared. Uncomment AngleResetViaIndxPin and comment AngleResetViaIndxPin_Dbug out.
2. **CCS**: Build the project: Debug project, Enable Silicon Real-time mode, Enable continuous refresh, and Resume.
3. **Processing**: Set a proper mode for position input by toggling positionInputMode. Setting the variable to 0 enables the direct-position input; while applying as 1 starts preprogrammed trajectory mode. When using the preprogrammed trajectory mode, make sure to load a correct path file. The path file should be in the same directory with the .pde file.
4. **Processing**: Execute the Processing_HardwareX.pde by pressing Run button. Check if Flag_enableSys is 1, and Flag_Run_Identify is 0 in the Real-Time Watch Window, Expressions tab.
5. **CCS**: Swing the arm so that the index-pin is detected while the arm travels. Detecting the index-pin initiates interrupt events for sciBRXISR() and timer0ISR() on the microcontroller. Also, Flag_Run_Identify becomes 1 in the Real-Time Watch Window, Expressions tab.
6. **CCS**: Wait until CtrlState and EstState become Online in the Real-Time Watch Window, Expressions tab.
7. **Processing**: Serial communication can be tested by whether or not the position data is logged without any missing bits. By default, a simple GUI should pop up after execution that displays the current joint angle in revolutions.
8. **Processing**: Move the arm to the position where the initial position input is programmed, and press the Enter key to enable the motor. Depending on the mode chosen in Section 5.3, exoskeleton will either guide the arm to the zero degrees position or apply the pre-programmed path. If the P gain is set too high or the inertia of the system is too low, the position error surges, resulting in the motor becoming unstable. However, please note that when a human is wearing the exoskeleton, the human arm provides additional damping to the system, and the proportional gains can be set to be much larger than if the exoskeleton is operating without a human inside. For the direct-position input, pressing up/down is programmed to increase/decrease the position by 0.01 rev.
9. **Processing**: Upon finishing the experiment, pressing the space bar key in the GUI terminates the Processing program and transmits a termination code to CCS. Both Flag_enableSys and Flag_Run_Identify become 0 in the Real-Time Watch Window, Expressions tab. This terminates timer0ISR() and motor control in CCS side, and Processing stores the logged data in a sub-folder, log/ under the installed folder.

## 7 Validation and characterization

**Fig. 14:**
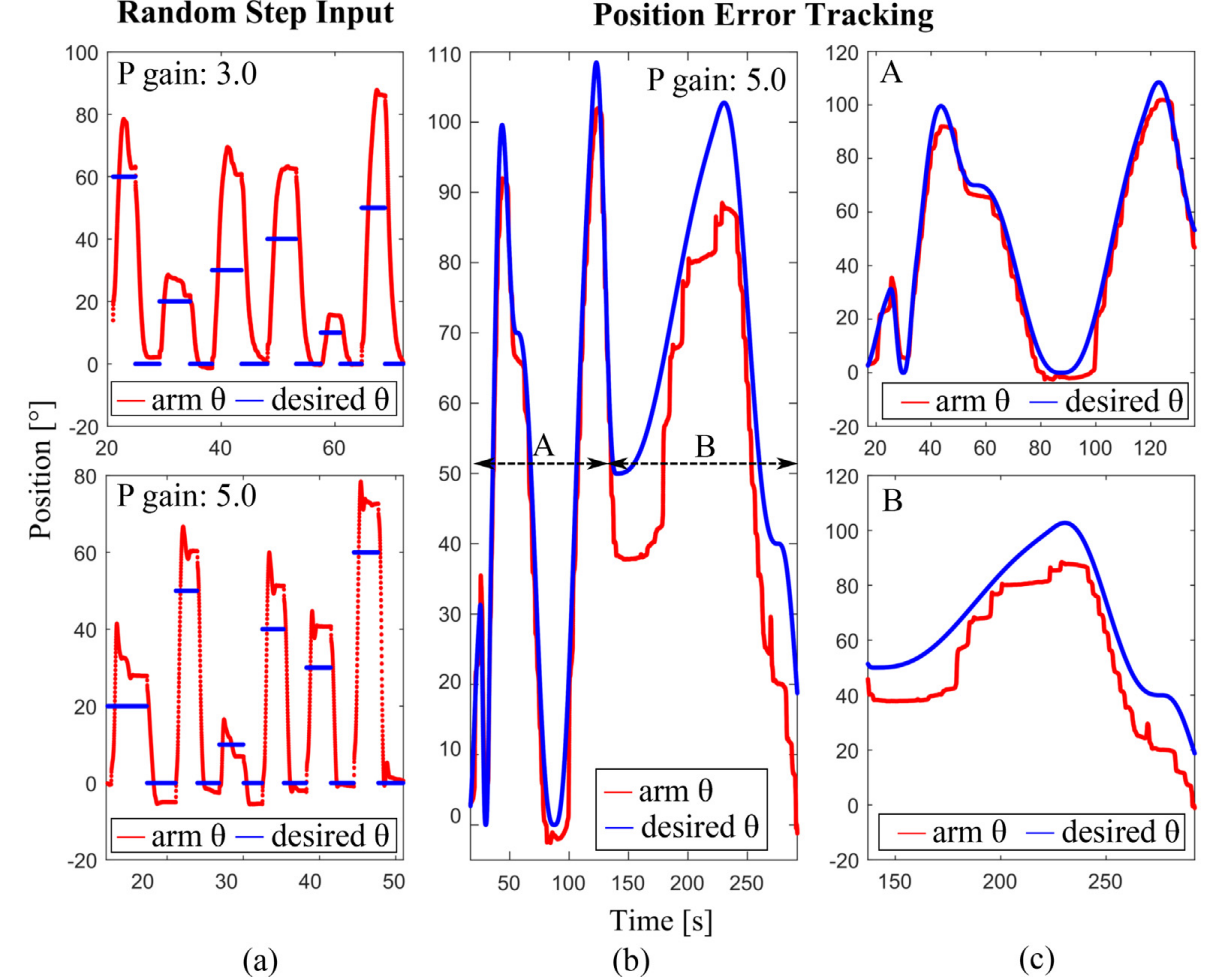
(a), Step responses with proportional gains (3.0 and 5.0). (b) and (c), Position tracking with kinesthetic guidance. (c) shows close-ups of different portions of (b). In all of the plots, the user’s arm angle is shown with the solid red line. The blue line indicates the input position.

Several different demonstrations of the system guiding a human’s arm are shown in Fig. 14. In each case, a position trajectory was transmitted from Processing to the PD controller in CCS. During the motion guidance, the exoskeleton applies a torque pushing the wearer toward the nominal position whenever the wearer deviates from that position. The subject was blinded during these tests and could not see their arm’s position. The tests were conducted with the wearer seated with their arm resting on a platform, as illustrated in Fig. 1.

In Fig. 14(a), proportional gains of 3.0 and 5.0 were each tested with six different step inputs ranging from 10–60°. The position inputs (blue lines) were applied in a random order via the exoskeleton, and the wearer tried to follow the applied torque (red lines). As can be seen, the wearer cannot perfectly follow the applied torque, and overshoots the desired final arm angle. The overshoots are larger when the gain is 3.0 (top) than 5.0 (bottom) because the wearer has difficulty differentiating between small positional differences when the proportional gain is low.

We also created a minimal-jerk trajectory, ranging from 0° to 100° (Fig. 14(b) and (c)). The red lines in these plots, which are the human arm angle, show a staircase pattern where the arm pauses for a moment followed by a more rapid motion to move toward the intended position. This trend is shown more distinctively in the part of the trajectory with a less-steep slope, B (Fig. 14(c), bottom plot) as compared to the A portion of the trajectory (Fig. 14(c), top). At the end of section A, the subject had an overshoot of approximately −12 degrees. They maintained a negative offset from the desired angle throughout section B. The tracking performance is determined by how well the subject perceives the kinesthetic cue of the external arm torque. Given that steeper slopes yield smaller position errors (A compared to B), we assume that a low rate of position change leads to poor perceptual resolution. When the desired position is slowly changing as in the B region, the wearer might have difficulty differentiating the torque change, leading to poor tracking performance. In general, we have observed that the test results can be affected by several factors, including: (1), how well the subject can focus on the arm torques; (2), how familiar the subject is with the test setup; (3), how tight the straps are; and (4), the types of foams and sleeve used in the exoskeleton.

While these results are very preliminary, they demonstrate the exoskeleton’s ability to be used as a motion training tool. This capability can be potentially used for rehabilitation or sports training, and the different hardware and software components can also be used separately to implement low-cost hobby motors for other actuator applications.

## 8 Human and animal rights

The study was approved by the Virginia Tech Institutional Review Board (IRB #16-175), and informed consent was obtained prior to the experiment.

## Declaration of Competing Interest

The authors declare that they have no known competing financial interests or personal relationships that could have appeared to influence the work reported in this paper.
