# Supplementary material for: An elbow exoskeleton for haptic feedback made with a direct drive hobby motor
Source: HardwareX. 2020 Oct 22;8:e00153. doi: 10.1016/j.ohx.2020.e00153 (PMC9041254; doi:10.1016/j.ohx.2020.e00153)
Supplement: Supplementary file 1 [file mmc1.pdf]

The Design Files are located at: http://dx.doi.org/10.17632/skm88ynyhv.4
